# Review of nonpharmacological interventions for delaying the effects of cerebral neuropathy caused by diabetes

**DOI:** 10.3389/fendo.2025.1621448

**Published:** 2025-08-27

**Authors:** Chi Lee, Chien-Wen Lai, Guan-Lin Wu, Shaoyu Yen, Chih-Mao Huang, Zong-Hsin Liu, Po-Chi Hu, Lun-De Liao

**Affiliations:** ^1^ Institute of Biomedical Engineering and Nanomedicine, National Health Research Institutes, Miaoli,, Taiwan; ^2^ Department of Biological Science and Technology, National Yang Ming Chiao Tung University, Hsinchu, Taiwan; ^3^ Metal Industries Research & Development Centre, Kaohsiung, Taiwan

**Keywords:** transcranial magnetic stimulation (TMS), diabetic peripheral neuropathy (DPN), neural inflammation, nonpharmacological interventions, neuromodulation

## Abstract

Diabetic peripheral neuropathy (DPN) is a debilitating complication of diabetes that affects nearly half of diabetic patients and manifests as chronic pain, sensory loss, and motor dysfunction. The limited efficacy of traditional pharmacological treatments, coupled with their side effects, has intensified the search for alternative therapies that not only mitigate symptoms but also delay the progression of diabetes-related neural complications. In this review, the potential of transcranial magnetic stimulation (TMS) as a nonpharmacological intervention for DPN is explored, with a focus on the ability of TMS to delay neural inflammation—a key factor in the progression of DPN—rather than directly treating diabetes. TMS has shown promising results in alleviating neuropathic pain, promoting nerve regeneration, and regulating autonomic nervous function, making it a strong candidate for delaying adverse neural outcomes. Other neuromodulation techniques, such as spinal cord stimulation (SCS), decompression nerve surgery (DNS), and transcranial direct current stimulation (tDCS), have also been examined for their efficacy in treating DPN. While TMS has significant therapeutic potential for protecting neural function and delaying inflammation, further research is needed to optimize treatment protocols and understand their long-term benefits. This review emphasizes the translational potential of neuromodulation technologies in delaying the progression of diabetes-induced neural damage, underscoring the need for further studies to translate these therapies into clinical practice.

## 1 Introduction

### 1.1 Diabetes and diabetic neuropathy

Diabetes mellitus (DM) is a common condition that has been prevalent for many years. The International Diabetes Federation (IDF) reported that in 2021, the global prevalence of diabetes in adults was 10.5% (1). The IDF suggested that this percentage will continue to rise. They reported that by the year 2045, 12.2% of people worldwide will be confirmed to have diabetes (1). Diabetes is a chronic metabolic disease caused by elevated blood glucose levels (2, 3). There are two possible etiologies of diabetes: a lack of insulin or insulin insufficiency (4, 5). Insulin is a hormone that facilitates the transfer of glucose from the blood into cells to further provide energy for metabolism (6–8). A lack of insulin or insulin insufficiency destroys this important process and may lead to diabetes.

The etiologies mentioned above can result in two main types of diabetes (9–11). Type 1 diabetes mellitus (T1DM) occurs when a patient’s own immune system antibodies attack and destroy insulin-producing β-cells in the pancreas, resulting in less insulin production (12, 13); however, it is unclear what exactly causes T1DM. Some findings have suggested that genetic or environmental factors are responsible for T1DM, whereas other findings have suggested that viruses could also play a role, as supported by case studies and blood test results (14–17). On the other hand, type 2 diabetes mellitus (T2DM) can be caused by either insufficient insulin production or insulin resistance (18–23). Research shows that a family history of T2DM increases an individual’s risk of developing the disease for genetic reasons (24–26). Furthermore, consuming high-fat foods, insufficient exercise, being overweight, being stressed, and having high blood lipid and cholesterol levels are factors that can cause T2DM (27). Studies have even shown that changes in the gut microbiota can be a cause of T2DM (28, 29). The gut microbiome is responsible for controlling fat accumulation and the progression of obesity-related diseases, making the gut microbiome a significant factor in T2DM development. When the microbiota of the gut is not regulated properly, a large amount of short-chain fatty acids are produced by bacteria, which can lead to impaired glucose metabolism and the occurrence of insulin resistance, ultimately resulting in T2DM (30, 31).

Both types of diabetes increase the glucose concentration in the blood. These changes can lead to several problems related to the circulatory system. Issues such as eye disease, kidney disease, and nerve damage can occur (32, 33). There is also a risk of severe circulatory system problems, such as heart disease, strokes, and diseases affecting blood flow in the limbs (34–36). Diabetic peripheral neuropathy (DPN) affects almost half of DM patients globally, making it a difficult, persistent challenge in DM treatment. DPN leads to problems such as lower limb pain and amputations, irregular heartbeats, and heart tissue breakdown, directly affecting everyday tasks and quality of life (37–39). Among patients with DPN, 20 to 30% also suffer from neuropathic pain (40, 41). This type of pain is hard to address because it usually gradually worsens and becomes more severe. To relieve pain, people often need medicines or other treatments (42, 43). The current understanding of DPN is based on abnormalities in glucose metabolism (44). A relatively high level of glucose triggers the activity of alternative metabolic pathways (45). These pathways and processes include the polyol and protein kinase C pathway, hexosamine and glucosamine formation, the accumulation of advanced glycation end products, and the anaerobic glycolytic process. One of these processes, alone or in combination, can lead to the beginning and progression of DPN (41, 45).

### 1.2 Drug treatment of diabetes and DPN

Presently, common therapeutic classes of drugs for diabetes include sulfonylureas, meglitinides, biguanides, peroxisome proliferator-activated receptor (PPAR) agonists, α-glucosidase inhibitors, glucagon-like peptide-1 (GLP-1) receptor agonists, dipeptidyl peptidase-4 (DPP-4) inhibitors, and sodium–glucose cotransporter 2 (SGLT2) inhibitors (46–49), as shown in **Table 1**. People with T2DM usually take sulfonylureas and meglitinides, which are insulin secretagogues. Insulin secretions facilitate the release of insulin by interacting with sulfonylurea receptors on pancreatic β-cells (50, 63, 64). Biguanides do not impact insulin release directly. The mechanism of action of biguanides involves decreasing the number of insulin-resistant cells. Biguanides also reduce the uptake of glucose in the intestine, which helps maintain glucose levels in the blood, prevents glucose delivery into the blood and facilitates the absorption of more glucose by cells (65, 66).

**Table 1:** Common drugs and their mechanisms of action in the pharmacological treatment of diabetes.

Type of drug	Mechanism of action	Reference
Sulfonylureas and repaglinide	Increase insulin secretion	(50)
Meglitinides	Enhance insulin response	(51)
Biguanides	Decrease hepatic gluconeogenesis Decrease peripheral insulin resistance	(52, 53)
PPAR agonist	Decrease peripheral insulin resistance Reduce fatty acids	(54, 55)
Alpha-glucosidase inhibitors	Slow absorption of carbohydrates	(56–58)
GLP-1 and DPP-4 inhibitors	Increase GLP-1 levels	(59, 60)
SGLT2 antagonists/inhibitors	Prevents reabsorption of glucose and increases the excretion of glucose in urine	(61, 62)

Commonly used drugs for diabetes treatment and their mechanisms of action, including sulfonylureas and repaglinide, meglitinides, biguanides, peroxisome proliferator-activated receptor (PPAR) agonists, alpha-glucosidase inhibitors, glucagon-like peptide 1 (GLP1) and dipeptidyl peptidase 4 (DPPIV) inhibitors, and sodium–glucose cotransporter 2 (SGLT2) antagonists/inhibitors, are listed.

PPAR agonists act as insulin sensitizers, activating transcription factors within the superfamily of hormone receptors, including PPARα, PPARγ, and PPARβ/δ. PPARs regulate metabolic functions and maintain energy stability, with PPARγ increasing cellular sensitivity to insulin and increasing glucose metabolism (54, 67, 68). Alpha-glucosidase inhibitors act by temporarily delaying the intestinal absorption of carbohydrates, resulting in a slower entry of glucose from food into the bloodstream, thereby inhibiting postprandial blood glucose levels (56, 69–71). Commonly used α-glucosidase inhibitors in clinical trials include acarbose, miglitol, and voglibose.

**Figure 1:**
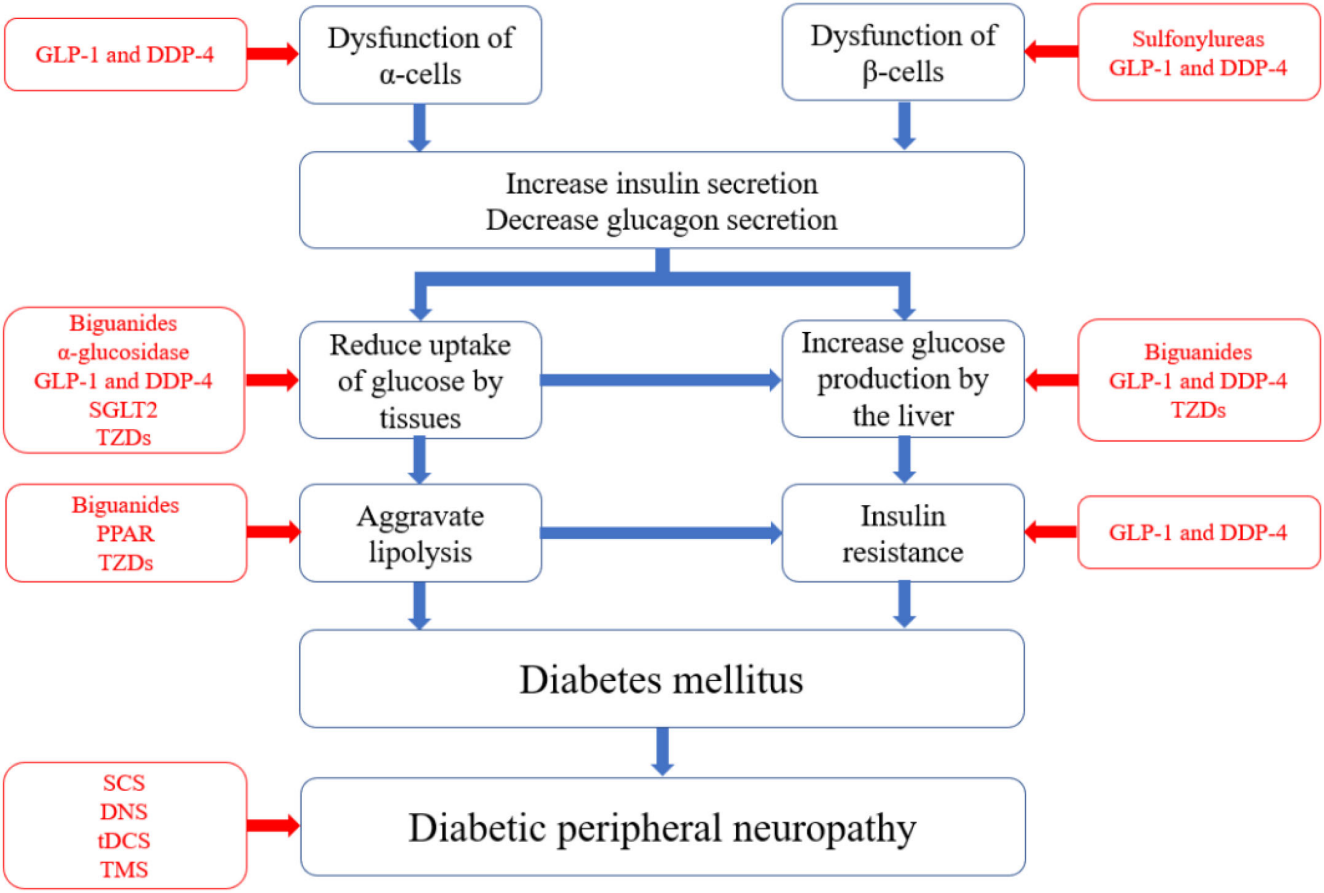
Pathophysiology of diabetes and corresponding pharmacological treatment. The blue boxes in the figure represent the pathological pathways of diabetes, starting from the dysfunction of α and β cells and leading to elevated blood glucose levels, insulin resistance, and ultimately diabetes. Each component can independently or synergistically contribute to the development and worsening of diabetes. The red boxes indicate the types of medications that target specific pathological pathways.

GLP-1 is a peptide consisting of 36 amino acids that plays crucial roles in various diabetes-related metabolic processes (72, 73). These roles include stimulating insulin secretion, lowering blood glucose levels, reducing gastric emptying, inhibiting food intake, and regulating rodent β-cell proliferation (59, 74, 75). DPP-4, a serine protease, degrades numerous peptides containing GLP-1 within biological organisms (60, 76). Therefore, the use of GLP-1 receptor agonists and DPP-4 inhibitors to increase GLP-1 levels and the half-life of GLP-1 in the body effectively lowers blood glucose levels (77, 78). SGLT2, which is found in the proximal convoluted tubule, is responsible for glucose reabsorption along with passive transport proteins, facilitated glucose transporters, and active cotransporter proteins (79). SGLT2 inhibitors prevent glucose reabsorption in the proximal convoluted tubule and promote its excretion in the urine, thereby reducing blood glucose concentrations (61, 80, 81). Various drugs are used to prevent diabetes. The different strategies are illustrated in **Figure 1**.

There are three classes of Food and Drug Administration (FDA)-approved drugs for treating DPN: pregabalin, duloxetine, and tapentadol. However, some experts challenge the approval of tapentadol because it is categorized as an opioid (82–84). Pregabalin binds to the α2δ subunit of calcium channels, reducing calcium influx into nerve endings and altering neurotransmitter release (85–87). Pregabalin is excreted primarily unchanged in the urine. Therefore, extra care is needed when administering pregabalin to people with kidney problems (88, 89). Duloxetine is a selective serotonin and norepinephrine reuptake inhibitor. It can cause side effects, including nausea, dizziness, and somnolence, when taken. Combining duloxetine with pregabalin, which has a different mechanism of action, has shown good clinical efficacy (90–92).

### 1.3 Nondrug treatment for diabetes and diabetic neuropathy

**Figure 2:**
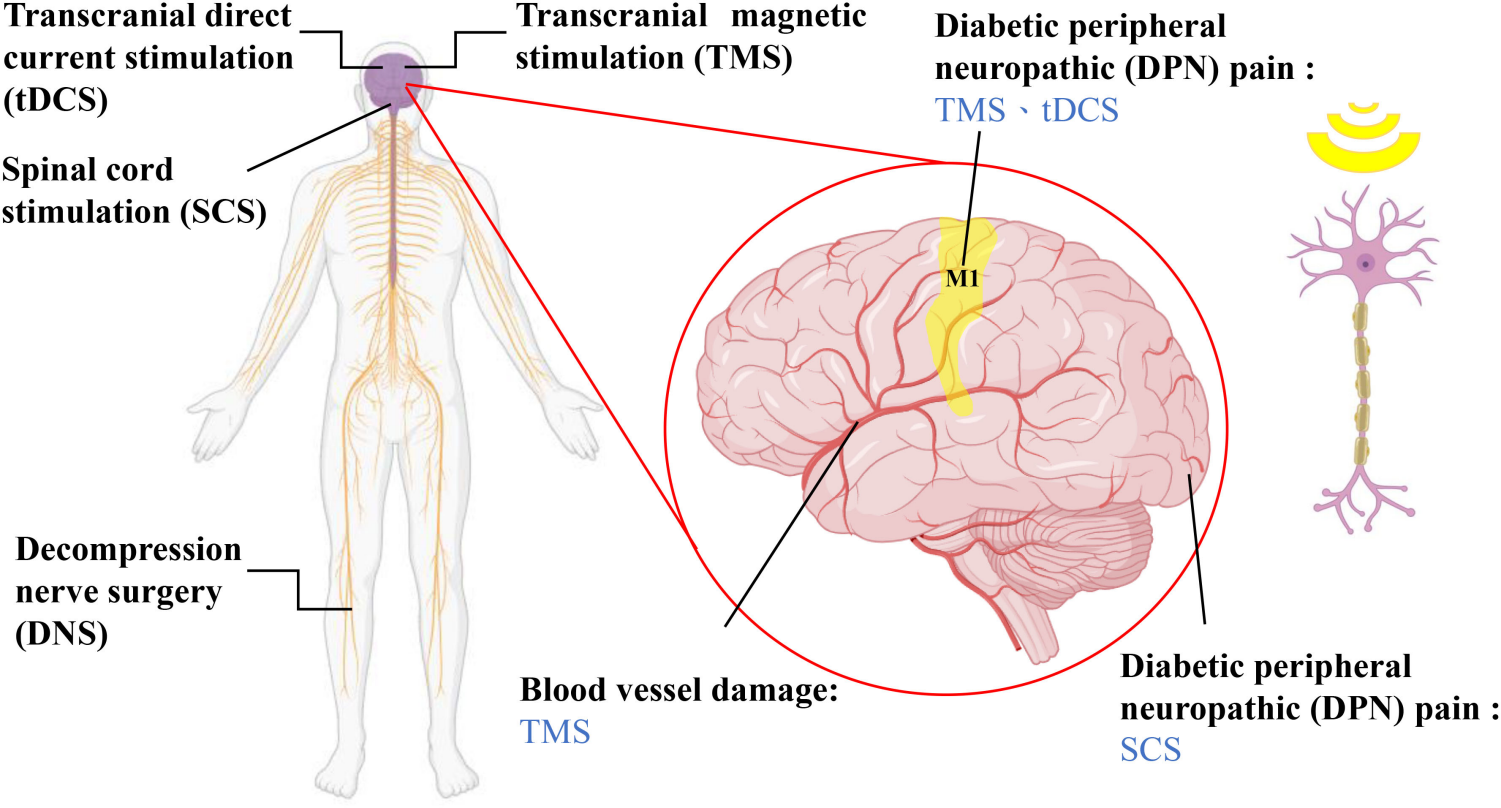
Nondrug treatments for diabetic neuropathy and the location of treatment. SCS involves placing electrodes on the dorsal side of the dura mater of the brain, utilizing fixed-frequency electrical stimulation to mask pain sensations (64). DNS is a surgical method that can alleviate chronic neuropathic pain caused by prolonged nerve compression in the lower limbs according to several observational studies, although its effectiveness has yet to be confirmed (56). tDCS and TMS are both noninvasive neuromodulatory techniques that can modulate neural functions in the brain, including through pain inhibition, depression control, and the relief of autonomic nerve dysfunction (74, 96, 100, 101). The magnified image on the right illustrates the brain regions that each therapy interferes with and the types of neuropathic conditions the therapies treat.

Medicines may help alleviate diabetes and DPN, but they also have notable side effects. We found that different nonpharmacological methods can be used to manage diabetes and DPN more safely. For example, diet control and exercise prevent people from developing diabetes (93–95). To alleviate and suppress DPN, methods such as spinal cord stimulation (SCS), decompression nerve surgery (DNS), transcranial direct current stimulation (tDCS), and transcranial magnetic stimulation (TMS) have also been used (96–99), shown as **Figure 2**.

TMS is a new treatment for DPN and has gained much attention because of research on the use of TMS for neuromodulation, nerve repair, and stimulation of neural pathways (102–104). TMS is a noninvasive method of localized cortical stimulation originally applied for central nervous system disorders (105, 106). However, by altering stimulation patterns and parameters, TMS can be used to assess the integrity of the CST and the degree of damage to motor conduction pathways (107, 108). Additionally, TMS can promote functional recovery after nerve injury by influencing brain blood flow and oxidative stress levels (109, 110). In TMS, a magnetic field is produced by altering the stimulation coil current to create an induced electrical field in the brain. When the current meets a certain limit, the axon hillock or interneurons are triggered. This shift in neuron responsiveness aids in adjusting brain activity (100). TMS is effective in treating different complications of diabetes, including painful conditions, depression, autonomic neuropathy, and vascular damage (102). However, as a relatively new therapy, limited research is available. Therefore, the aims of this review are to summarize the current research on the use of TMS in the treatment of DPN and compare DPM with other nonpharmacological treatment modalities.

### 1.4 Research motivation and objectives

Despite the availability of numerous pharmacological and nonpharmacological strategies for the management of diabetes and its complications, DPN remains a prevalent and debilitating condition that significantly impairs patients’ quality of life. Current pharmacotherapies for DPN are often associated with limited efficacy, adverse effects, and poor long-term outcomes, whereas conventional nonpharmacological approaches yield inconsistent therapeutic benefits. These limitations underscore the urgent need for alternative modalities that are both effective and well tolerated. In recent years, TMS, a noninvasive neuromodulation technique, has emerged as a promising treatment option for various neurological and pain-related disorders. Preliminary investigations suggest that TMS may exert beneficial effects on DPN through mechanisms involving cortical reorganization, pain modulation, and neurovascular regulation. However, evidence supporting its application in DPN remains fragmented, and its efficacy relative to that of other nonpharmacological interventions has not been systematically examined. Therefore, this review aims to synthesize the current literature on the use of TMS in treating DPN, evaluate its therapeutic potential in comparison with other nonpharmacological modalities, and elucidate the underlying mechanisms that may contribute to its clinical effects. This review aims to address an important gap in existing studies and inform future research directions and clinical approaches for managing diabetic neuropathy.

## 2 Exercise and diet in the treatment and management of diabetes

### 2.1 Mechanisms of action of alleviation and treatment methods and approaches for controlling diabetes in the diet

#### 2.1.1 Impact of diet on diabetes

A high-fat diet serves as a standard method to induce obesity in animal models. Excessive intake of saturated fats leads to the overexpansion of adipocytes in adipose tissue, resulting in hypoxia. Hypoxia triggers a cascade of responses, including the activation of hypoxia-inducible factor 1 (HIF-1) gene expression. HIF-1, a transcription factor, becomes activated in low-oxygen environments, leading to the overexpression of other proteins, such as c-Jun N-terminal kinase and IκB kinase. In turn, inflammatory responses are induced in cells (111). Inflammation typically manifests as the production of proinflammatory cytokines, which further exacerbate the inflammatory process (111, 112). Inflammation causes a chain reaction in cells, which leads to difficulty in insulin regulation. Insulin usually controls fat breakdown by reducing the influence of hormone-sensitive lipases within fat-storing cells. However, when these cells resist insulin, stored fats are transferred into free fatty acids (113), move into the bloodstream and are taken up by various organs. This process further causes these organs to resist insulin and leads to T2DM (101, 111, 113).

#### 2.1.2 Dietary approaches for the prevention and alleviation of diabetes

It is clear that what we eat greatly impacts early diabetes development. Eating properly, such as choosing low-fat foods, limiting carbohydrates, or following a ketogenic diet, can effectively prevent and manage diabetes (111, 113, 114). A healthy diet involves the consumption of daily essential nutrients, such as water, protein, fat, carbohydrates, and other micronutrients, to maintain overall health. According to the World Health Organization recommendations for a healthy diet, individuals should consume 400 grams of fruits and vegetables per day, as well as legumes, grains, and nuts. The fat content should be kept below 30% of the total caloric intake, and the intake of simple sugars should be limited to less than 10% of the total caloric intake. Daily caloric intake should be set on the basis of individual energy expenditure (115).

In contrast to a high-fat diet, a low-fat diet restricts the consumption of fats, including cholesterol (116). By reducing the risk of obesity and insulin resistance, a low-fat diet helps prevent diabetes (117–119). For diabetes patients, reducing fat intake can effectively alleviate the development of insulin resistance in organs. A carbohydrate-restricted diet (CRD) involves the replacement of high-carbohydrate foods with higher protein- or fat-containing foods and fiber-rich vegetables (120–122). CRD can effectively control blood sugar levels, and studies suggest that reducing carbohydrate intake can lower the risk of developing T2DM (120–123). Therefore, for patients who are unresponsive to medications that lower glucose levels, a diet limited in carbohydrates is a practical choice. For example, a ketogenic diet causes the body to produce ketones, similar to when the body is fasting (124). This type of diet makes the body favor ketones over glucose for energy (124, 125). Ketogenic diets have garnered significant popularity in contemporary times and are increasingly employed for the management of diabetes. An intriguing aspect of a ketogenic diet is its ability to effectively reduce blood glucose levels (126, 127). Research findings indicate that individuals following a ketogenic diet may require only half the amount of insulin needed before initiating this dietary regimen (124, 128, 129). For example, a four-month research study revealed a decrease in antidiabetic medicine use with a ketogenic diet. Some patients could even stop using antidiabetic medications entirely (130). As a result, experts view a ketogenic diet as a tool that can help regulate blood glucose, assisting those with diabetes.

### 2.2 Mechanisms of action of exercise in the treatment of diabetes and exercise modalities

#### 2.2.1 Impact of exercise on diabetes

Exercise and diet control are ways to lower weight. Losing weight with activity and less caloric intake can help approximately 80% of T2DM patients (93–95). Furthermore, regular exercise can alleviate inflammatory symptoms in muscle cells, reduce cellular insulin resistance, and stimulate the activity of adenosine monophosphate-activated protein kinase (AMPK). In turn, AMPK activity facilitates the translocation of glucose transporter 4 (GLUT4) to the cell membrane, contributing to the control of blood glucose levels (131–133).

#### 2.2.2 Exercise modalities for the prevention and alleviation of diabetes

Among all exercise modalities, aerobic exercise, resistance training, and high-intensity interval training (HIIT) are the most common. Aerobic exercise involves rhythmic activities, such as walking and running, which involve large muscle groups (134–136). A minimum of 150 minutes per week of moderate to vigorous aerobic exercise can increase VO_2_max cardiac output (134, 137). The risks of heart issues and death are greatly lower in patients with T2DM who exercise (138). Additionally, aerobic exercise helps balance fat and other body-building substances. Furthermore, exercise lowers hemoglobin A1c (HbA1c) levels (139, 140). In contrast to the fat-reducing effects of aerobic exercise, resistance training focuses more on altering the structure of the body’s muscle tissue through the use of weight machines, free weights, and resistance bands (141). Resistance training improves health indicators such as bone density, blood pressure, blood lipids, insulin sensitivity, and muscle strength by 10–15% (142–144). This effect not only slows the progression of diabetes in elderly patients but also contributes to a reduction in the risk associated with other diseases.

As a popular workout style, in HIIT, individuals push their limits in short, intense workouts of approximately 10 minutes (145, 146). HIIT is a useful method for controlling blood fat and glucose levels (147, 148). HIIT even increases muscle power and the response to insulin (147–149). One HIIT workout is short and might be a good fit for T2DM patients willing to take on a challenge. However, HIIT is not suitable for everyone. Some experts believe that combined aerobic and resistance workouts could work better (150). This combined workout regimen could lead to more positive results in terms of insulin response and HbA1c reduction than either type alone (151–153). The efficacy of a combined exercise strategy in terms of reducing the risk of cardiovascular diseases has been demonstrated (153, 154). In conclusion, a mixed aerobic and resistance regimen could be suitable for T2DM patients for whom HIIT is too difficult.

### 2.3 Limitations of exercise and diet in the treatment and management of diabetes

While exercise and dietary interventions are widely acknowledged as foundational nonpharmacological approaches for managing type 2 diabetes, their effectiveness is subject to several important limitations. One key constraint is the influence of concurrent antidiabetic medications, such as metformin, SGLT2 inhibitors, or insulin, which can alter baseline glycemic levels and potentially obscure or exaggerate the true effects of lifestyle changes (155, 156). Additionally, individual behavioral and lifestyle factors, including smoking, alcohol use, chronic stress, poor sleep quality, and prolonged sedentary behavior, can negatively impact insulin sensitivity and systemic inflammation, thereby confounding the outcomes of exercise- and diet-based interventions.

## 3 Diabetic neuropathy alleviation and treatment

### 3.1 Spinal cord stimulation

In the past, it was usually difficult to efficiently ease pain with the standard medication for DPN (43, 157). This standard treatment is often associated with substantial side effects and low tolerance (158, 159). To reduce our dependence on medicines, the search for different treatment methods or adjunct therapies is now an important part of research. Compared with traditional medicine, SCS provides pain relief that lasts eight times longer (160). SCS is a method in which the sense of pain is replaced with sensory paresthesia. The electrodes are placed on the dorsal surface of the spinal cord. Then, a certain strength of electrical current is sent through the spinal cord and masks the sensation of pain (161, 162). With technological advancements, SCS is no longer limited to the original 40~60 Hz low-frequency stimulation; a unique waveform at 10 kHz can be utilized to alleviate the pain associated with DPN without inducing sensory disturbances. The evidence suggests that, following high-frequency SCS stimulation, 87.5% of patients experienced at least a 50% reduction in pain during a six-month follow-up (163). Furthermore, more than half of patients experienced a reduction in pain intensity of over 50%, even five years after treatment (164). The supporting evidence confirms the effectiveness of the SCS.

### 3.2 Decompression nerve surgery

DPN is characterized by symptoms of regular, long-term nerve pressure, including discomfort, a burning sensation, a pin-and-needle sensation, and a loss of sensation (165, 166). Because of these parallels, some studies have suggested that nerve compression causes DPN (167). The affected areas in the human body include three nerves located in the legs, including the distal fibula on the lateral side of the lower leg, the intersection of the leg with the posterior heel on the medial side, and the junction of the short extensor muscle of the thumb with the branch of the deep peroneal nerve (168). These nerves are often compressed because of the narrow tunnel they pass through at these locations. Thus, DNS is widely used to prevent pain due to DPN (99, 169), but the effectiveness of DNS is still a matter of debate. Indeed, most researchers have proposed the effectiveness of DNS simply on the basis of observations (99). Future research is needed to further clarify the feasibility and efficacy of DNS in the treatment of DPN.

### 3.3 Transcranial direct current stimulation

#### 3.3.1 Mechanism of action of tDCS

tDCS is a noninvasive brain stimulation technique, and its efficacy was confirmed in rodents several decades ago (170). Anodal tDCS can increase cortical excitability, whereas cathodal stimulation can reduce cortical excitability (171, 172). The effects of tDCS are rapid, with cortical excitability typically being influenced within seconds of stimulation, and prolonged stimulation can significantly extend the duration of cortical modulation (173–175). Rather than inducing changes in only single brain cells, tDCS has a greater impact on whole-brain networks (176). Several cells in our bodies, such as endothelial cells, immune cells, and brain cells, are sensitive to changes in electrical fields. The activities of these cells are manipulated when tDCS is applied (177). These findings indicate that tDCS can influence the development of certain diseases by affecting these cells.

#### 3.3.2 Application of tDCS in DPN

tDCS successfully lowers pain in people suffering from DPN-related discomfort. tDCS adjusts brain activity, specifically in the left dorsolateral prefrontal cortex (DLPFC) and the primary motor cortex (M1) (178). Anodal stimulation of M1 can inhibit thalamic activity, thereby regulating the pain threshold and intensity (179). The DLPFC is one of the most commonly activated brain regions during pain episodes (180). Therefore, disrupting the involvement of the DLPFC in pain perception through electrical stimulation can significantly alleviate patients’ pain. Additionally, stimulation of regions such as the medial prefrontal cortex, anterior insula, anterior cingulate cortex, and bilateral amygdala can reduce negative emotions such as anxiety and depression (181). The use of tDCS to alleviate pain associated with DPN has been considered feasible in numerous studies (182). However, the impact of tDCS remains inconsistent. The effectiveness of tDCS can be affected by age, genetic factors, medicines, lifestyle factors, and even physical activities. Moreover, even the same patient can experience different results at different time points (183). Determining the right level of stimulation for each patient can be challenging when tDCS is applied in clinical practice.

### 3.4 Transcranial magnetic stimulation

#### 3.4.1 Principles and mechanism of action of TMS

TMS, a noninvasive cortical modulation technique, was initially employed for functional localization in the central nervous system (109). Over time, with changes in coil shape, stimulation patterns, and other parameters, TMS is now being utilized for treating central and peripheral neuropathologies, evaluating the activity of the corticospinal tract, and determining the extent of damage to motor functions and conduction pathways (184, 185). Additionally, TMS can influence local cerebral blood flow to alter oxidative stress levels, promoting functional recovery in the lower limbs following nerve injury (109).

**Figure 3:**
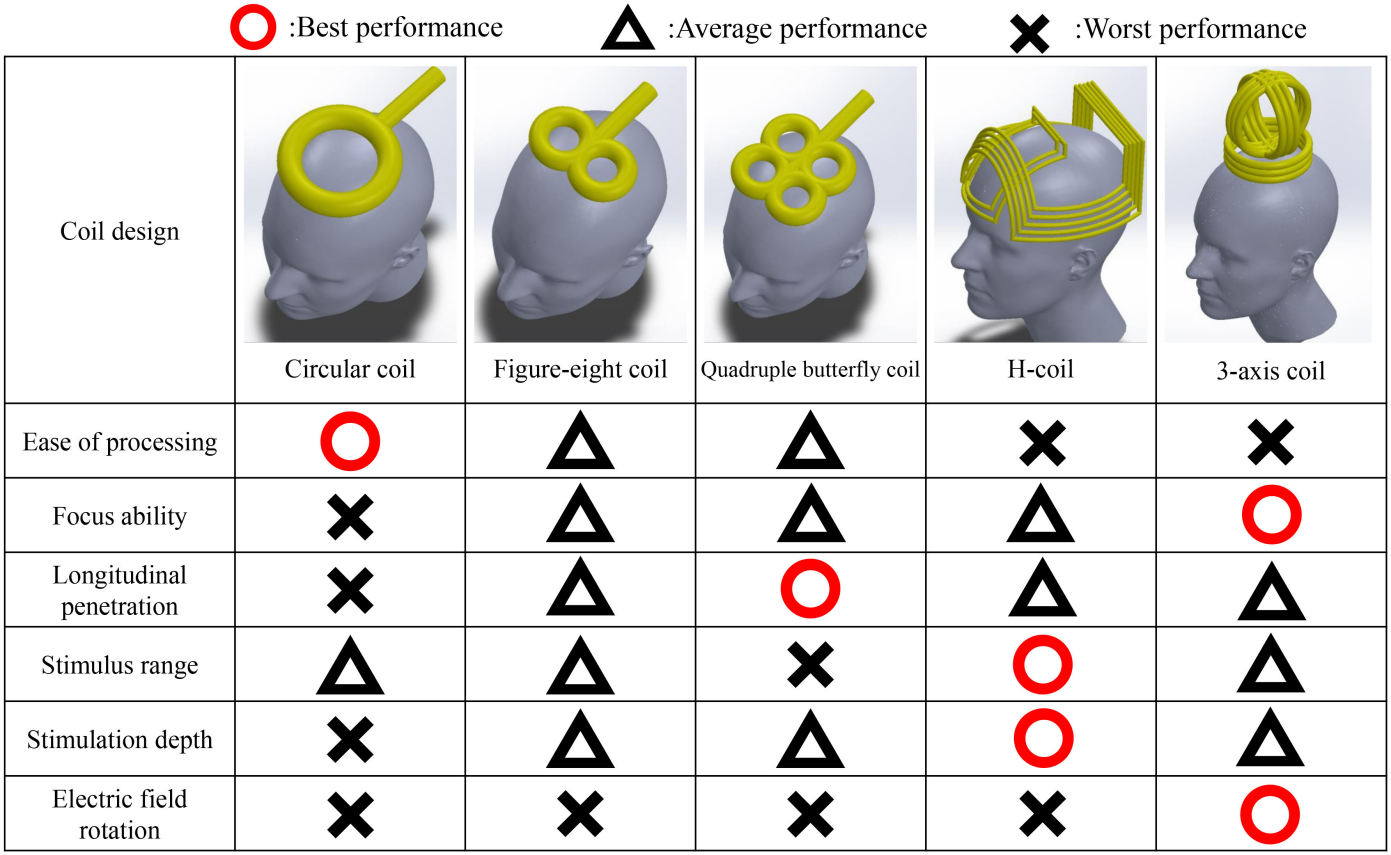
Performance comparison of coil designs. Since the development of the TMS, the design of the coil has been improved many times. Currently, four coil designs are most commonly used (188, 189), each with distinct advantages and disadvantages, as illustrated in the figure. The circular coil was the earliest design and is relatively easy to manufacture, although it lacks precision and depth in stimulation (190–193). The figure-8 coil was developed as an improvement over the circular coil, enhancing both the focus and penetration depth (194–196). The quadruple butterfly coil, optimized for increased stimulation depth along a vertical axis, provides the highest focus and penetration depth among the four designs but covers a relatively narrow area (197, 198). Finally, the H-coil, in contrast to earlier designs, is considered capable of achieving greater stimulation depth and coverage owing to its skull-encompassing structure, allowing for stimulation across a wider range of brain regions (199, 200). The 3-axis coil provides a stimulation range similar to that of traditional circular coils but overcomes the difficulty in achieving focal stimulation. By adjusting the current output of the three coil sets, this coil enables rotation of the electric field to finely tune the stimulation location (201).

In a TMS, magnetic field pulses are created via electrical currents following Faraday’s law. These shifting magnetic fields pass through the skull to affect the intended area, producing currents in the process (186). When the intensity of electrical current flow increases to a certain level, nerves in the focus area are stimulated. TMS adjusts neuron signals and modulates brain activity (100, 187). The intensity of the magnetic field follows the inverse square law, so the intensity decreases rapidly with depth. To stimulate deeper areas of the brain, a stronger intensity is required at the surface (188). To address this, various coil shapes have been developed, including circular, figure-eight, quadruple butterfly, and Hesed coils (H-coils) (188, 189), as shown in **Figure 3**. The determination of coil shape depends on the specific brain region area and depth. Circular coils are characterized by their simple design and ease of manufacturing (190, 191). However, the circular electric field it generates cannot achieve focused stimulation. Although techniques have been developed to focus the electric field by altering the winding angle and density of the coil (192, 193), its focusing capability remains inferior to that of other coil designs. In comparison, figure-8 coils, which consist of two adjacent circular coils, can generate a focused electric field at the intersection point beneath the coils (194, 195), allowing for deeper and more precise stimulation than circular coils. Compared with figure-eight coils, quadruple butterfly coils, which are derived from figure-eight coils, are composed of four circular coils and are more commonly used for stimulating long fiber structures (197, 198). Finally, the more novel H-coils, compared with the aforementioned designs, are larger in size and have a more complex winding pattern. This structure allows the electric field to penetrate deeper into the brain with a slower rate of field attenuation (199, 200), although this comes at the cost of reduced focusing ability. In addition, the TMS pattern includes single-pulse TMS, paired-pulse TMS, and repetitive pulse TMS (rTMS) (202). This parameter affects the results of the TMS as well. Single-pulse TMS is commonly used for specific cortical site stimulation because of its effectiveness in measuring the cortical response to each pulse (187, 203). Paired-pulse TMS employs two paired pulses to stimulate the same or different cortical areas, serving to assess functional connectivity between two regions (204). rTMS induces cortical effects through a continuous sequence of pulses, allowing control over the produced effects and duration through the adjustment of stimulation intensity, frequency, and time (205, 206). TMS can be used for long-lasting, high or low activation or blockage of central and peripheral nerves. This allows focused stimulation of connections between brain areas. To date, encouraging results of TMS have been shown for diagnosing and treating central and peripheral nerve injuries. Even though the use of TMS in DPN patients is not widespread, TMS still offers significant promise as a treatment option.

#### 3.4.2 Value of TMS in diagnosing DPN

DPN originates in the early stages of diabetes, as elevated blood glucose levels following dietary intake lead to the onset and subsequent progression of DPN (207). Therefore, American Diabetes Association (ADA) scholars emphasize DPN screening in patients exhibiting early symptoms of diabetes (208). In the early phase of DPN, smaller nerve groups are usually targeted first, which could affect pain sensation. However, because small and large nerve fibers work collaboratively, clear signs of this nerve damage might not be observable. Thus, DPN examinations should cover both nerve types to better identify small nerve damage (207). According to the ADA 2022 Clinical Practice Guidelines, the recommended protective sensory testing methods can detect only severe sensory loss (209). Hence, more accurate early screening methods are needed for a comprehensive assessment. The TMS is likely to assist in addressing these related issues.

TMS alone is not sensitive enough to serve as a standalone technique for evaluating DPN. However, TMS can be utilized to detect motor-evoked potentials when combined with other assessment methods, such as functional magnetic resonance imaging (fMRI), electroencephalography (EEG), and Doppler ultrasound monitoring (210, 211). This integrated approach can help establish potential connections between various functional parameters, aiding in a more comprehensive evaluation and diagnosis of DPN patients (212, 213). Combined with the above methods, TMS can be used to detect motor-evoked potentials, which are crucial for assessing a patient’s neural and muscular function. Through TMS, key parameters such as nerve conduction velocity, excitability thresholds, and response times can be observed, facilitating the identification of abnormalities in the nervous system (109, 210). In addition, fMRI provides details about brain activity and connections. These findings reveal how the nervous system processes pain and sensory information in diabetes patients (186, 210). EEG detects changes in brain electrical activity related to pain perception, which is crucial for evaluating central nervous system abnormalities in DPN patients. Doppler ultrasound, which is used to monitor vascular health and focuses on blood flow velocity and microcirculation, is essential for diagnosing vascular or nerve lesions in DPN patients. TMS can stimulate specific brain regions, and when combined with fMRI, neural circuit responses can be observed in real time; when combined with EEG, fMRI can synchronously record changes in brain electrical activity; and when combined with Doppler ultrasound, fMRI can assess blood flow changes in response to neural stimulation. This multimodal approach helps to comprehensively assess and diagnose nerve and vascular abnormalities in early-stage DPN patients. Therefore, the TMS plays a significant role in this multifaceted assessment process.

#### 3.4.3 Application of TMS in neural protection and regeneration

In patients with DPN, the lower extremities typically manifest as the initial site of neuropathic changes, progressing from sensory impairments to the emergence of motor symptoms as the condition worsens (214). The main pathological pathways of DPN in the presence of elevated blood glucose levels include the polyol, hexosamine, and protein kinase C pathways, as well as oxidative stress and inflammatory responses that act on neural origins, all of which contribute to impaired nerve function (215). Studies have shown that, after 8–12 weeks of a high-fructose diet, C57BL/6 N mice exhibit significant hippocampal neuroinflammatory responses; these responses activate glial cells and astrocytes, leading to neuroglial proliferation and a substantial decrease in the number of hippocampal neurons and newborn neurons (216). Matrix metalloproteinases (MMPs), which are downstream effectors of hyperglycemia and oxidative stress, play dual roles in extracellular matrix remodeling and neuroregeneration. Under controlled activation, MMPs facilitate axonal sprouting and the structural reorganization necessary for nerve regeneration by promoting the degradation of inhibitory matrix components and enabling the migration of neural progenitor cells (217).

On the basis of the aforementioned mechanisms, TMS coils have been used in several studies to generate magnetic field pulses and stimulate the heads of rats with gray matter injuries. This approach can restore the regenerative capacity of neurons in the hypothalamic region (218). Research has demonstrated that rTMS promotes remyelination in demyelinated white matter regions by increasing the proliferation, migration, and differentiation of oligodendrocyte precursor cells (219). Moreover, evidence suggests that rTMS can increase the expression of brain-derived neurotrophic factor, which in turn activates the TrkB signaling cascade—a critical pathway involved in synaptic plasticity, axonal growth, and the survival of injured neurons (220–222). These findings collectively indicate that TMS, as a neuromodulatory technique, can promote neural regeneration.

#### 3.4.4 TMS for alleviating neuropathic pain in DPN patients

Various approaches have previously been used to treat neuropathic pain caused by DPN, including SCS, DNS, tDCS, or conventional pharmaceutical interventions; however, several clinical challenges are still faced with these treatments. Issues such as discomfort during treatment, a pain relief rate less than 50%, and significant side effects associated with drug therapy have been reported (223). TMS is able to directly stimulate the thalamus, which serves as the pain integration center, through cortical brain activity. When stimulated, the thalamus blocks the sensory transmission pathway from the spinal cord to the thalamus, reducing the patient’s sensitivity to pain (224). Research findings indicate that for chronic pain resulting from spinal cord injury, low-frequency (1-Hz) stimulation of the M1 region of the brain can inhibit cortical excitability, thereby increasing the pain threshold and alleviating pain perception (225). Stimulation of the M1 region has also been shown to mitigate abnormal temperature perception caused by chronic neuropathic pain (226). Research has shown that the use of an H-coil for high-frequency (20 Hz) stimulation of deeper and broader areas is effective in relieving lower limb pain in patients (227). Additionally, focused stimulation of the lower limb motor cortex region via a figure-8 coil can reduce pain sensitivity in patients over a period of five weeks (228). The analgesic efficacy of rTMS in DPN has been supported by multiple studies involving varying designs and stimulation protocols. As summarized in **Table 2**, both animal and human studies have reported significant reductions in pain scores and the levels of inflammatory markers, with some trials reporting reductions of up to 60% in allodynia or subjective pain intensity. These outcomes highlight the therapeutic potential of rTMS; however, given the diversity in coil types, stimulation frequencies, and cortical targets employed across studies, further investigations are warranted to optimize stimulation parameters and identify patient-specific predictors of responses.

**Table 2:** Summary of the pain relief outcomes of rTMS in DPN and neuropathic pain.

Participants	Frequency	Stimulation region	Coil type	Duration	Pain assessment tool	Pain relief outcome	Statistical significance	Reference
Animals (NP rat model)	1 Hz	M1	figure-8 coil	8 days	Behavioral scale, cytokine assays	~60% reduction in mechanical allodynia; decreased IL-1β and TNF-α expression	*P*< 0.05	(225)
Animals (CCI rat model)	10 Hz	PFC	circular coil	4 weeks	PWMT, ELISA (IL−1β, IL−6, TNF−α, TRPV1)	Significantly increased PWMT; reduced IL−1β, IL−6, TNF−α, TRPV1 in PFC and spinal cord	*P*< 0.05	(229)
Animals (NP rat model)	0.5 Hz	Amygdala	butterfly coil	8 days	Mechanical and thermal threshold, Golgi stain, gene/protein assays	Increased pain thresholds; reversed dendritic spine deficits; decreased integrin αvβ3, P2×7R, NLRP3 signaling	*P*< 0.05	(230)
15 patients with chronic neuropathic pain	10 Hz	left M1	figure-8 coil	1 day	VAS, thermal perception tests	Decrease in the mean VAS score from 7.8 to 4.5; improved thermal detection	*P*< 0.03	(226)
18 patients with painful diabetic neuropathy	20 Hz	M1	H-coil	5 days	VAS, RIII reflex area, BDI	Decrease in the VAS score to below 70% of baseline; decrease in the RIII reflex area by ~30%	*P*< 0.01	(227)
30 patients with refractory DPN	10 Hz	lower limbs motor cortex	figure-8 coil	5 days	Numeric rating scale	Decrease in the NRS score from 6.5 ± 0.9 to 3.6 ± 0.7 (after 1 day) and to 5.3 ± 1.1 (after 1 week)	*P*< 0.01	(228)

The table highlights stimulation parameters, pain assessment tools, and reported pain reduction outcomes, including both subjective and objective indicators.

#### 3.4.5 TMS for treating depression induced by DPN

Depression is an emotional disorder characterized by features such as loss of pleasure, despair, intensified guilt, and physical distress (231). The pathological mechanisms underlying this psychological illness are complex and involve responses to stress, neural structure and function, and immune–neurological imbalances, all of which are associated with dysregulation of the hypothalamic–pituitary–adrenal (HPA) axis (232). The hippocampus, a crucial regulator of stress responses, undergoes neuronal damage or apoptosis due to HPA axis dysregulation induced by hyperthyroidism (233). TMS has been demonstrated to significantly improve mood, cognition, motor function, and sensation (221, 234). TMS also influences neurotransmitters and the endocrine response of the HPA axis in patients with depression (235). Therefore, the use of TMS to restore HPA axis homeostasis and prevent hippocampal neuronal apoptosis has become a therapeutic strategy for treating depression. The feasibility of this approach has been acknowledged in studies using high-frequency (10 Hz) TMS in Sprague–Dawley rats (236). Additionally, research findings suggest that peripheral neuropathic pain caused by diabetes is a factor contributing to depression. Consequently, the use of TMS to inhibit M1 and the descending pain system to treat muscle-related pain may have an adjunctive effect on diabetes-induced depression (237). Although the mechanisms underlying diabetes-induced depression remain unclear, TMS is practical for modulating depressive symptoms under high-glucose conditions. TMS can also be employed to assess the efficacy of antidepressant medications in patients, serving as an auxiliary therapeutic tool.

#### 3.4.6 Application of TMS in the treatment of diabetic autonomic neuropathy

Diabetic autonomic neuropathy (DAN) affects the cardiovascular, gastrointestinal, and urogenital systems, with approximately 20% of diabetic patients experiencing DAN, often accompanied by other peripheral neuropathies (238). Autonomic neuropathy occurring in the cardiovascular system may lead to tachycardia, hypotension, dizziness, and motor impairment, even resulting in a loss of control over the sympathetic and parasympathetic nerves of the heart (239). When the gastrointestinal tract is affected, symptoms such as esophageal dysmotility and gastroparesis may result. DAN can also disrupt the urogenital system, causing recurrent urinary tract infections, pyelonephritis, and bladder and sexual dysfunction (240).

Currently, the understanding of how the cerebral cortex controls autonomic nervous system function remains uncertain. However, studies have shown that the use of TMS to stimulate the sympathetic nervous system can induce transient changes in the cardiovascular system (241). In another study, TMS was utilized to stimulate M1, and the neural connection between the brain and kidneys was demonstrated. The findings indicated a significant increase in urinary protein levels in both diabetic and nondiabetic individuals after stimulation, suggesting a potential link between the brain and renal autonomic functions (242). While the ability of TMS to influence the autonomic nervous system has been demonstrated, further research is needed to determine whether such effects can have therapeutic benefits for patients.

#### 3.4.7 TMS for addressing vascular damage in DPN patients

Cardiovascular diseases and microvascular complications contribute to the increased incidence and mortality of diabetes patients (243). DPN induces endothelial cell proliferation in blood vessels, leading to thickening of the capillary basement membrane. These structural changes result in narrowed blood vessels, reducing the blood supply to neuronal fibers (244). Among these, the nerve–vascular barrier and oxygen tension of dorsal root nerves and other peripheral nerve trunks are lower, increasing their susceptibility to microvascular changes (245). The dorsal root nerves, which act as a system to regulate blood flow, are adversely affected when damaged, disrupting blood flow regulation and exacerbating the process of vascular damage. Endothelial dysfunction can also trigger neuropathies; in diabetes, hyperglycemia-induced adhesion of endothelial cells or thrombus formation can lead to blood vessel obstruction, causing ischemic damage to neuronal fibers (246).

Studies have shown that TMS can induce angiogenesis and significantly increase cerebral blood flow (247). Additionally, stimulation of brain regions with low-frequency (1-Hz) TMS increases oxygen consumption and metabolic rates by approximately 28%, which is correlated with increased blood flow (248). Although there is currently no research on the use of TMS to modulate vascular blood flow and endothelial cell proliferation in DPN patients, future studies in this area are highly anticipated.

## 4 Conclusion and future challenges

DPN affects approximately 50% of diabetic patients and results in chronic pain, sensory loss, and motor dysfunction. Although approved by the FDA, traditional pharmacological treatments, such as pregabalin and duloxetine, provide limited relief for approximately 20–30% of patients and are often accompanied by significant side effects. This necessitates the exploration of alternative therapeutic approaches, particularly nonpharmacological interventions. In this review, the potential of TMS as a noninvasive treatment for delaying neural inflammation and mitigating the symptoms of DPN is highlighted. TMS has shown promise in reducing pain sensitivity by up to 50% over several weeks of treatment and has demonstrated potential in promoting nerve regeneration and regulating autonomic nervous function. As outlined in **Table 3**, the efficacy of TMS is influenced by various stimulation parameters, such as frequency and intensity, with low-frequency (1 Hz) stimulation being effective in reducing cortical excitability and alleviating pain, whereas high-frequency (10 Hz) stimulation shows benefits in mood regulation. The flexibility of coil designs, including figure-eight and H-coils, further expands the application of TMS across different neuropathic conditions.

**Table 3:** TMS frequencies and stimulation intensities selected in various research areas.

Research direction	Type	Frequency and intensity	Duration	Reference
Neuroregeneration	LF, HF	1 Hz, 120%RMT 20 Hz, 120%RMT	3 days	(249)
Neuropathy	LF-rTMS	1 Hz, 200mT	8 days	(225)
Depression	HF-rTMS	10 Hz, 50%RMT	15 days	(236)
Oxidative stress	HF-rTMS	10 Hz, 120%RMT	2 days	(250)
Autonomic nervous function	HF	20 Hz, 90%RMT	1 day	(251)

The transcranial magnetic stimulation (TMS) patterns used in research studies for different diseases are listed. Low frequency (LF) represents low frequency, high frequency (HF) represents high frequency, and the resting motor threshold (RMT) describes the output intensity used in the studies.

Other neuromodulation techniques, such as SCS, have demonstrated an 87.5% success rate in reducing pain intensity by at least 50% after six months of treatment, with pain relief being maintained in 55% of patients five years posttreatment. Similarly, DNS and tDCS present alternative therapeutic options, although their clinical applications remain limited owing to inconsistent outcomes and a lack of large-scale clinical trials. In contrast, TMS is a noninvasive intervention with a relatively low incidence of adverse effects. Reported side effects include transient headaches, scalp discomfort, and, in rare cases, seizures, particularly in individuals with a history of epilepsy or those undergoing prolonged high-frequency stimulation. To integrate these therapies into routine clinical practice, large-scale, randomized controlled trials are needed to establish the long-term efficacy, safety, and cost-effectiveness of TMS and other neuromodulation approaches. Moreover, advanced diagnostic tools such as fMRI and EEG could assist in the personalization of these treatments, enabling more precise and effective interventions for DPN patients. **Table 3** outlines the various frequencies and intensities used in TMS for different neurological conditions, emphasizing the need for the standardization of these parameters to optimize patient outcomes. As the global prevalence of diabetes is projected to rise to 12.2% by 2045, addressing the challenges of DPN through innovative therapies such as TMS will become increasingly important. While neuromodulation techniques, especially TMS, show significant therapeutic potential, the application of TMS in the treatment of DPN remains relatively limited, and comprehensive treatment protocols and long-term data are still lacking. In light of the multifactorial nature of DPN, and considering that the effectiveness of rTMS may vary significantly with factors such as age, gender, and cortical thickness, integrative approaches that combine rTMS with existing pharmacological, nutritional, or exercise-based interventions should be explored in future research. For example, combining rTMS with agents that enhance neuroplasticity or reduce inflammation may yield synergistic effects. Similarly, coupling rTMS with aerobic or resistance training, both of which are known to improve glucose metabolism and nerve function, may help optimize therapeutic outcomes. These multimodal strategies represent promising directions for developing personalized and noninvasive treatments that address both the neurological and metabolic aspects of diabetic neuropathy. The broader implications of this work suggest that TMS could serve as a complementary tool in clinical neurology and diabetes care, particularly for patients who are unresponsive to pharmacological treatments. The integration of TMS into multidisciplinary care plans could enhance treatment personalization, reduce long-term healthcare costs associated with diabetes complications, and potentially prevent the progression of neuropathic symptoms in high-risk populations. In conclusion, while TMS and other neuromodulation techniques represent promising frontiers in the nonpharmacological treatment of DPN, optimizing treatment protocols and expanding clinical trials will be key to the broader adoption of these techniques. With the increasing burden of diabetes worldwide, advancing these therapies could significantly improve the quality of life of millions of diabetic patients suffering from neuropathy.
